# Identification of interaction partners using protein aggregation and NMR spectroscopy

**DOI:** 10.1371/journal.pone.0270058

**Published:** 2022-09-09

**Authors:** Young Kee Chae, Han Bin Shin, Tae Rin Woo

**Affiliations:** Department of Chemistry, Sejong University, Seoul, Korea; Nanyang Technological University, SINGAPORE

## Abstract

The interaction among proteins is one of the most fundamental methods of information transfer in the living system. Many methods have been developed in order to identify the interaction pairs or groups either *in vivo* or *in vitro*. The *in vitro* pulldown/coprecipitation assay directly observes the protein that binds to the target. This method involves electrophoresis, which is a technique of a low resolution as well as a low throughput. As a better alternative, we wish to propose a new method that is based on the NMR spectroscopy. This method utilizes the aggregation of the target protein and the concomitant signal disappearance of the interacting partner. The aggregation is accomplished by the elastin-like polypeptide, which is fused to the target. If a protein binds to this supramolecular complex, its NMR signal then becomes too broadened in order to be observed, which is the basic phenomenon of the NMR spectroscopy. Thus, the protein that loses its signal is the one that binds to the target. A compound that interferes with these types of bindings among the proteins can be identified by observing the reappearance of the protein signals with the simultaneous disappearance of the signals of the compound. This technique will be applied in order to find an interaction pair in the information transfer pathway as well as a compound that disrupts it. This proposed method should be able to work with a mixture of proteins and provide a higher resolution in order to find the binding partner in a higher throughput fashion.

## Introduction

With the development and advancement of sequencing methods, such as Next Generation Sequencing (NGS), the genomes of the organisms are being revealed more rapidly, but the function of the protein or RNA encoded by the DNA sequence is still often unknown [1, 2]. For this reason, the progress of proteomics is very slow, and the information about their interaction partners is more often unknown even if the functions of the proteins are known [3, 4]. The interaction network of these proteins is collectively called the interactome, which is similar to systems biology [5]. The most widely used protein-protein interaction investigation methods so far include the yeast two hybrid *in vivo*, GST pull-down *in vitro*, and the fluorescence or luminescence imaging *in vivo* or *in vitro* [6, 7]. Each method has its limitations as well as merits, and other methods have been devised and developed in order to overcome them [8].

Protein-protein interactions play an important role in a number of complex processes that range from the simple formation of heterologous/homologous protein multimers to cell-to-cell signaling, the regulation of enzyme activity, and the regulation of DNA replication [9]. The information flow can be controlled in the living organisms by inhibiting or promoting this interaction. In particular, it is expected that treatment or a cure will become possible by regulating the interaction of the disease-related proteins [10]. The common cold, the flu, or COVID-19 viruses can enter the human body through protein-protein interactions [11]. Therefore, developing a high-resolution technology that is capable of discriminating a specific interaction more rapidly and in a high throughput fashion would be desirable and also necessary.

The elastin-like polypeptide (ELP) is a synthetic biopolymer that undergoes a reversible aggregation at the transition temperature [12]. The ELP has many repeats of a pentapeptide motif, Val-Pro-Gly-X-Gly, where X is any amino acid [13]. The transition temperature where the ELP aggregates varies depending on the identity of X, the number of repetitions of the pentamer, and the salt concentration [14]. When the ELP module is fused to the target protein, the reversible aggregation property is preserved, with a small change in the transition temperature, so a method for a simple separation of the fusion protein by centrifugation has been proposed [15]. It has been reported that the ELP is linear or intrinsically disordered below the transition temperature, and it assumes the form of spherical aggregates above the transition temperature [16]. The reversible aggregation property of the fusion protein can be applied to tissue scaffolding, drug delivery, and metal recovery [17–19].

NMR spectroscopy is a versatile tool that is used in order to observe the molecular structure or dynamics in the solution or the solid state [20, 21]. NMR provides invaluable information that ranges from the verification of the synthesized organic compound to the structure elucidation of the macromolecules, such as proteins or DNA. NMR spectroscopy has an intrinsic limitation despite the versatility. In order to be able to produce an NMR signal, the molecule of interest should tumble fast enough. If the tumbling rate decreases in the solution, the signal becomes broader, which disappears in extreme cases. In most NMR applications, this is a hurdle that needs to be overcome, but we take advantage of it in this proposal. We make the signals from the molecule of interest disappear if it binds to the target. In our previous report, we successfully materialized this concept in order to observe the disappearance of the signal of the binding ligand [22]. We wish to expand the application in order to probe the interaction between two proteins and its disruption by a ligand by observing disappearance and reappearance of the signals from the protein of interest. The research concepts we have constructed are as follows.

### Aggregation of the partner protein by the target-ELP fusion protein

**Fig 1:**
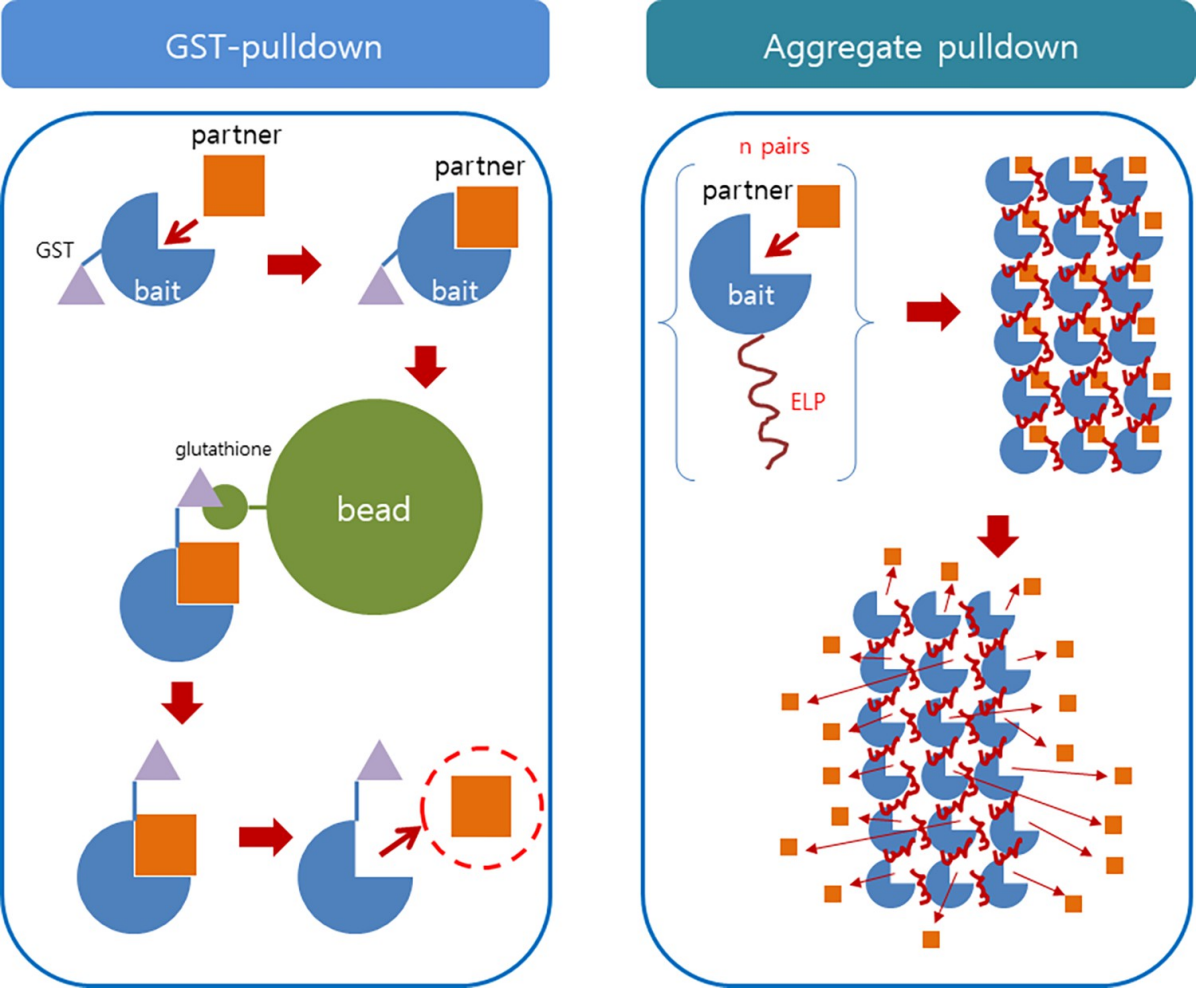
Comparison of the search process for the partner proteins that bind to the target proteins. The well-established GST-pulldown method (left). The GST-fused bait protein is immobilized to the glutathione bead to pick up a partner protein that binds to it. The proposed aggregate pulldown (right). The ELP-fused bait protein aggregates above the transition temperature, and forms a solid support where the partner protein is immobilized by binding to the bait. By increasing the salt concentration, the partner protein is released to the solution phase while the bait-ELP still remains in the aggregate state.

The ELP (Elastin-Like Polypeptide) makes a reversible aggregation depending on the temperature or the salt concentration. It can induce the same aggregation in the fusion protein state, which is illustrated in Fig 1. This aggregation may precipitate into a solid phase depending on the concentration. If the bead used in the GST pulldown is macroscopic, the aggregate of this method can be regarded microscopic, so it can still be soluble. Using the vector constructed in our laboratory, the plasmid that is required in order to fuse the target protein with the ELP can be readily prepared, which is expected to facilitate the proposed research [23].

### Observation of disappearance/restoration of NMR signal through the aggregate formation/dissociation

**Fig 2:**
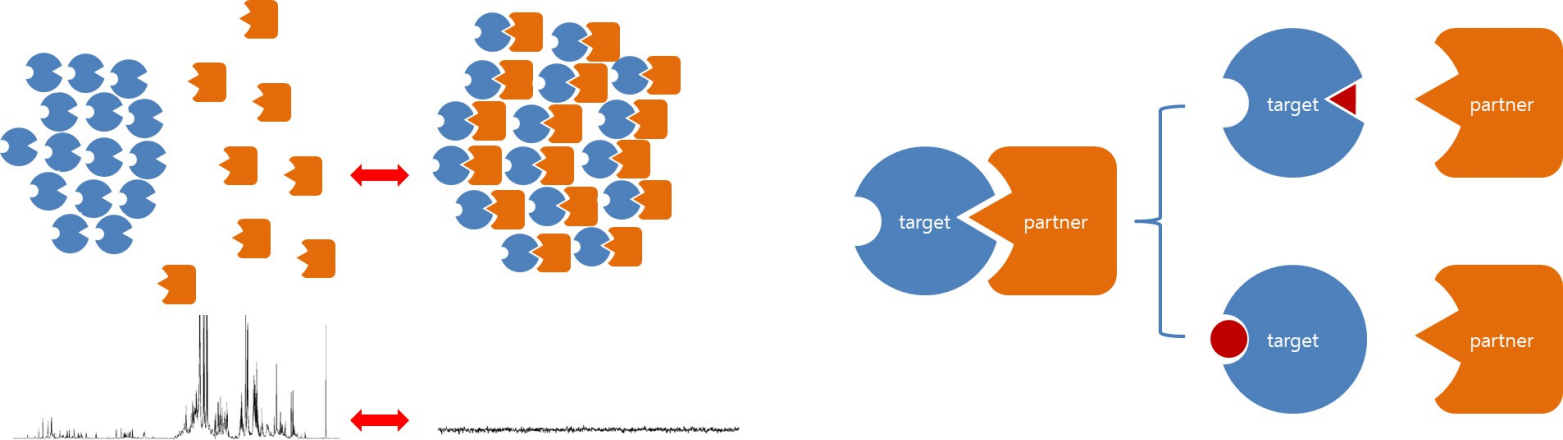
Disappearance and restoration of the NMR signals by the reversible aggregation. The NMR signals originate from the partner protein, which is colored orange (left). Interaction inhibitors: competitive or non-competitive mechanisms (right). The ELP domain is not shown, but it is assumed that the ELP is aggregated into a solid phase by binding to one another. The NMR signal of the partner protein will disappear when it binds to the aggregate. The inhibitor will disrupt the binding of the partner and the target proteins. Note that the NMR spectrum in this figure is not a real data, but employed only to represent the disappearance/reappearance of the signals.

As mentioned above, since the NMR signal disappears when the size of the object to be observed becomes very large, it is expected that the signal of the partner protein will disappear if it is bound to the target protein that forms an aggregate [24]. When a general ionic compound or a specific substance that interferes the binding is added, the partner protein is released and transferred to a solution state. As a result, the NMR signal is expected to be restored, which is illustrated in Fig 2 (left) The disappearance/reappearance of these signals is reversible.

### Target-partner separation by interaction inhibitors

**Fig 3:**
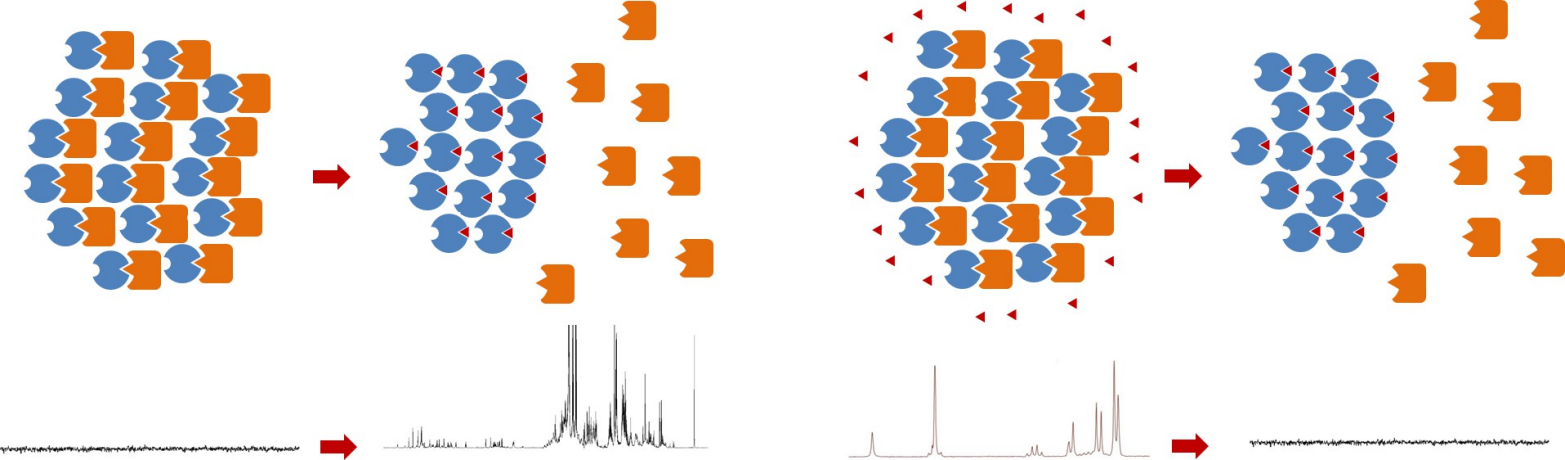
NMR spectral changes. The signals of partner protein is restored as it is released from the target (left). The signals of the inhibitor will disappear as it binds to the target (right). Note that the NMR spectrum in this figure is not a real data, but employed only to represent the disappearance/reappearance of the signals.

It is expected that the NMR signals of the two proteins will disappear when the target-partner co-aggregates above the transition temperature, which is illustrated in Fig 2 (left). When a compound that interferes with the interaction is added, the target remains in the aggregate. However, the partner protein is released and transferred to the solution phase, and its NMR signal is expected to be restored, which is shown in Fig 3 (left). Therefore, the type of compound that restores the NMR signal can be regarded an inhibitor of the target-partner interaction, which would be via a competitive or non-competitive mechanism in this method (Fig 2, right). It is also possible that two or more compounds bind to the protein complex at the same time, which occurs randomly or sequentially. It will be possible to readily determine whether there are inhibitor candidates in the added mixture by observing the restoration of the NMR signal. Reciprocally, the signals from these types of inhibitors will disappear if they bind to the target-ELP aggregates (Fig 3, right).

## Materials and methods

### Target-partner selection

We chose the RNase S-peptide and S-protein as a test pair, which is known to assemble in order to form RNaseS [25]. The two fragments are small enough to be NMR-observable by themselves, but their signals are expected to disappear above the transition temperature by tethering one or the other to the ELP module. For the second pair, we chose the MBP (maltose binding protein) and the periplasmic domain of Tar, which is a chemoreceptor, or the loop P2 of MalF, which is a subunit of the maltose transport system [26, 27]. The ELP we have chosen is I48 whose transition temperature is around 298K. The aggregation should be ensured if the NMR data is collected at 303K.

### Protein production and purification

The gene of the target protein-ELP fusion protein and the gene of the partner protein are inserted into pVP65KR or pVP65KR-ELP, which was constructed in our laboratory [23]. Using Rosetta2(DE3)pLysS as a host, the bacterial cultures will be grown in an autoinduction medium in order to simplify production. After the cells are disrupted, the proteins will be purified using a Ni-NTA column.

### Metabolite extraction

After culturing and harvesting *E*. *coli* and freeze-drying, the hot water extraction will be used in order to denature all of the inherent enzymes and to extract the metabolites [28, 29]. Ultrafiltration will be performed in order to further increase the purity of the low-molecular-weight metabolites. The filtrate will be lyophilized.

### NMR spectroscopy

1D ^1^H NMR experiments will be employed [30, 31]. The previous experience suggested that the 1D noesy (noesypr1d, Bruker pulseprogram) was quite suitable for the detection [22]. Fig 3 illustrates the process where the partner protein is released and the signal is restored (left), and the signal of the inhibitor disappears (right) as the inhibitor binds to the target protein. The binding strength may vary, but the partner protein can remain unobservable by using the ELP-fused target protein in excess. As a result, the concentration of the free partner protein will be kept low. Data will be collected from the ELP module alone as a negative control, as well as from the partner protein alone (without the ELP tag) as a positive control.

## Results and discussion

The purpose of this study is to develop a method in order to identify an interaction pair as well as a binding inhibitor by observing/unobserving the NMR signal of a partner protein, which is based on a reversible aggregation/dissolution. This method can be regarded a nano-size version of the GST-pulldown. Artefacts can arise due to the ELP module, but whenever a fusion system is employed, the conformation of the protein of interest can be affected. However, if the linker between the target protein and ELP is long enough, we believe that it is reasonable to assume that they behave independently. By comparing with the negative and positive controls as mentioned in the previous section, we will be able to minimize the artefact.

The research will proceed from proving our concept with a couple of known interaction pairs, then to identifying/extracting a partner protein from a protein mixture, and finally to identifying a binding inhibitor from a mixture of small molecules. The first step is the main focus of this protocol. After this step is successfully completed, this method can be expanded to probing the protein library, which we aim as the second step. The protein library can be constructed from the total proteins of *E*. *coli*. The protein library will be mixed with the target, and the binding proteins will be separated by centrifugation above the transition temperature. The mixture of the binders will go through the chromatographic separation procedure, and each binder can be identified using the mass spectrometry (HPLC-MS), which is widely used in the proteomics [32]. Each binder will be tested by using NMR spectroscopy as mentioned above. We speculate that this method can be further developed to a high-throughput screening of the target protein by using NMR in the future, although we expect this will take a while. In this conceptual scheme, the protein library will be mixed with the target in order to make an NMR sample as above, and the two H-1 spectra will be collected both below and above the transition temperature, which will produce the H-1 NMR difference spectrum. Deconvoluting the spectrum against those in the NMR database, which is an essential prerequisite which we hope will be constructed in the future, will help identify the binders. Currently, there are several NMR data repositories around the world, and the data are freely available [33]. With the NMR chemical shift and integral data of the proteins, the above mentioned difference spectra can be analyzed in a similar way that the metabolite spectrum is analyzed by the Chenomx software [34]. The final step involves the identification of a binding inhibitor. As for the observation of the restoration of the protein signals by an inhibitor (Fig 3), if the ratio of the inhibitor to the target protein is kept well below 1, then almost all the inhibitors will stay bound to the target, and its signal will be unobservable. On the other hand, the partner protein will be released to the solution phase, and their signals will become observable. Because we are looking for a signal which turns observable by an inhibitor, even a simple 1D ^1^H NMR may suffice. The 1D version of noesy experiment was known to suppress the solvent signal effectively [35]. However, if it becomes too difficult to observe the protein signal in case the ligand mixture is used (instead of one inhibitor), the partner protein will be labeled with ^13^C and/or ^15^N, and the 2D [^1^H-^13^C] or [^1^H-^15^N] HSQC experiments will be performed for higher sensitivity and resolution [18]. The ligand mixture will be prepared from the metabolites of *E*. *coli*, and it can be labeled with ^13^C. The metabolite mixture will play a role as a small molecule library. As for the observation of the disappearance/restoration of the ligand signals, it is possible to identify which signals have disappeared upon adding the partner-target aggregate. The 2D HSQC will become necessary in this process as the spectrum of the ligand mixture will undoubtedly become very complex.

The discovery and elucidation of the protein-protein interactions is a very important example of the recently-discovered novel coronavirus (nCoV) [36]. The mechanism of action is that the coat protein of this virus interacts with the human lung cell surface protein and penetrates into the cell, which thereby induces a pneumonia [37, 38]. This virus as well as rhinovirus and influenza, which cause the cold or the flu, and viruses that cause SARS, MERS, or HIV bind to proteins on the target cell’s surface and penetrate into the cells [39]. Therefore, the accurate identification of these types of protein-protein interactions is an essential prerequisite for the systematic development of the inhibitors or therapeutics. We hope that this study will provide a unique platform to the goal of finding a lead compound.

## References

[1] Giani AM, Gallo GR, Gianfranceschi L, Formenti G. Long walk to genomics: History and current approaches to genome sequencing and assembly. Computational and Structural Biotechnology Journal. 2020;18:9–19. doi: 10.1016/j.csbj.2019.11.002 31890139 PMC6926122

[2] Harrow J, Nagy A, Reymond A, Alioto T, Patthy L, Antonarakis SE, et al. Identifying protein-coding genes in genomic sequences. Genome Biology. 2009;10(1):201. doi: 10.1186/gb-2009-10-1-201 19226436 PMC2687780

[3] Levy SE, Myers RM. Advancements in Next-Generation Sequencing. Annual Review of Genomics and Human Genetics. 2016;17(1):95–115. doi: 10.1146/annurev-genom-083115-022413 . 27362342

[4] Ang MY, Low TY, Lee PY, Wan Mohamad Nazarie WF, Guryev V, Jamal R. Proteogenomics: From next-generation sequencing (NGS) and mass spectrometry-based proteomics to precision medicine. Clinica chimica acta; international journal of clinical chemistry. 2019. 10.1016/j.cca.2019.08.010 31421119

[5] Vidal M, Cusick Michael E, Barabási A-L. Interactome Networks and Human Disease. Cell. 2011;144(6):986–98. doi: 10.1016/j.cell.2011.02.016 21414488 PMC3102045

[6] Brückner A, Polge C, Lentze N, Auerbach D, Schlattner U. Yeast two-hybrid, a powerful tool for systems biology. Int J Mol Sci. 2009;10(6):2763–88. doi: 10.3390/ijms10062763 . 19582228 PMC2705515

[7] Einarson MB, Pugacheva EN, Orlinick JR. GST Pull-down. CSH Protoc. 2007;2007:pdb.prot4757. Epub 2007/01/01. doi: 10.1101/pdb.prot4757 . 21357137

[8] De Las Rivas J, Fontanillo C. Protein–protein interaction networks: unraveling the wiring of molecular machines within the cell. Briefings in Functional Genomics. 2012;11(6):489–96. doi: 10.1093/bfgp/els036 22908212

[9] Braun P, Gingras A-C. History of protein–protein interactions: From egg-white to complex networks. PROTEOMICS. 2012;12(10):1478–98. doi: 10.1002/pmic.201100563 22711592

[10] Ruiz C, Zitnik M, Leskovec J. Identification of disease treatment mechanisms through the multiscale interactome. Nature Communications. 2021;12(1):1796. doi: 10.1038/s41467-021-21770-8 33741907 PMC7979814

[11] Brito AF, Pinney JW. Protein-Protein Interactions in Virus-Host Systems. Frontiers in microbiology. 2017;8:1557–. doi: 10.3389/fmicb.2017.01557 . 28861068 PMC5562681

[12] Varanko AK, Su JC, Chilkoti A. Elastin-Like Polypeptides for Biomedical Applications. Annual Review of Biomedical Engineering. 2020;22(1):343–69. doi: 10.1146/annurev-bioeng-092419-061127 . 32343908

[13] Kowalczyk T, Hnatuszko-Konka K, Gerszberg A, Kononowicz AK. Elastin-like polypeptides as a promising family of genetically-engineered protein based polymers. World J Microbiol Biotechnol. 2014;30(8):2141–52. Epub 2014/04/04. doi: 10.1007/s11274-014-1649-5 . 24699809 PMC4072924

[14] Christensen T, Hassouneh W, Trabbic-Carlson K, Chilkoti A. Predicting transition temperatures of elastin-like polypeptide fusion proteins. Biomacromolecules. 2013;14(5):1514–9. Epub 2013/04/08. doi: 10.1021/bm400167h . 23565607 PMC3667497

[15] Hassouneh W, Christensen T, Chilkoti A. Elastin-like polypeptides as a purification tag for recombinant proteins. Current protocols in protein science. 2010;Chapter 6:Unit-6.11. doi: 10.1002/0471140864.ps0611s61 . 20814933 PMC3076942

[16] Roberts S, Dzuricky M, Chilkoti A. Elastin-like polypeptides as models of intrinsically disordered proteins. FEBS letters. 2015;589(19 Pt A):2477–86. Epub 2015/08/29. doi: 10.1016/j.febslet.2015.08.029 . 26325592 PMC4599720

[17] Glassman MJ, Avery RK, Khademhosseini A, Olsen BD. Toughening of Thermoresponsive Arrested Networks of Elastin-Like Polypeptides To Engineer Cytocompatible Tissue Scaffolds. Biomacromolecules. 2016;17(2):415–26. Epub 2016/01/20. doi: 10.1021/acs.biomac.5b01210 . 26789536 PMC4752000

[18] Rodríguez-Cabello JC, Arias FJ, Rodrigo MA, Girotti A. Elastin-like polypeptides in drug delivery. Advanced Drug Delivery Reviews. 2016;97:85–100. doi: 10.1016/j.addr.2015.12.007 26705126

[19] Hussain Z, Kim S, Cho J, Sim G, Park Y, Kwon I. Repeated Recovery of Rare Earth Elements Using a Highly Selective and Thermo-Responsive Genetically Encoded Polypeptide. Advanced Functional Materials. n/a(n/a):2109158. doi: 10.1002/adfm.202109158

[20] Felli IC, Brutscher B. Recent Advances in Solution NMR: Fast Methods and Heteronuclear Direct Detection. ChemPhysChem. 2009;10(9–10):1356–68. doi: 10.1002/cphc.200900133 19462391

[21] Ashbrook SE, Griffin JM, Johnston KE. Recent Advances in Solid-State Nuclear Magnetic Resonance Spectroscopy. Annual Review of Analytical Chemistry. 2018;11(1):485–508. doi: 10.1146/annurev-anchem-061417-125852 . 29324182

[22] Chae YK, Um Y, Kim H. A simple and sensitive detection of the binding ligands by using the receptor aggregation and NMR spectroscopy: a test case of the maltose binding protein. Journal of Biomolecular NMR. 2021. doi: 10.1007/s10858-021-00381-x 34524563 PMC8441238

[23] Chae YK, Kim H. Development of an Autoinducible Plasmid for Recombinant Protein Production. Protein & Peptide Letters. 2021;28:1–10. doi: 10.2174/0929866528666211105113750 34749604

[24] Foster MP, McElroy CA, Amero CD. Solution NMR of large molecules and assemblies. Biochemistry. 2007;46(2):331–40. doi: 10.1021/bi0621314 . 17209543 PMC2596980

[25] Watkins RW, Arnold U, Raines RT. Ribonuclease S redux. Chem Commun (Camb). 2011;47(3):973–5. Epub 2010/11/16. doi: 10.1039/c0cc03864d . 21079871 PMC3077719

[26] Zhang Y, Gardina PJ, Kuebler AS, Kang HS, Christopher JA, Manson MD. Model of maltose-binding protein/chemoreceptor complex supports intrasubunit signaling mechanism. Proceedings of the National Academy of Sciences of the United States of America. 1999;96(3):939–44. doi: 10.1073/pnas.96.3.939 . 9927672 PMC15329

[27] Licht A, Schneider E. ATP binding cassette systems: structures, mechanisms, and functions. Central European Journal of Biology. 2011;6(5):785. doi: 10.2478/s11535-011-0054-4

[28] Chae YK, Kim SH, Markley JL. Relationship between recombinant protein expression and host metabolome as determined by two-dimensional NMR spectroscopy. PLOS ONE. 2017;12(5):e0177233. doi: 10.1371/journal.pone.0177233 28486539 PMC5423636

[29] Chae YK, Kim SH, Um Y. Relationship between Protein Expression Pattern and Host Metabolome Perturbation as Monitored by Two-Dimensional NMR Spectroscopy. Bull Korean Chem Soc. 2019;40(7):634–41. doi: 10.1002/bkcs.11743

[30] Araç D, Murphy T, Rizo J. Facile detection of protein-protein interactions by one-dimensional NMR spectroscopy. Biochemistry. 2003;42(10):2774–80. Epub 2003/03/12. doi: 10.1021/bi0272050 . 12627942

[31] Zondlo NJ. SAR by 1D NMR. J Med Chem. 2019;62(21):9415–7. doi: 10.1021/acs.jmedchem.9b01688 31663734

[32] Noor Z, Ahn SB, Baker MS, Ranganathan S, Mohamedali A. Mass spectrometry–based protein identification in proteomics—a review. Briefings in Bioinformatics. 2020;22(2):1620–38. doi: 10.1093/bib/bbz163 32047889

[33] Baskaran K, Craft DL, Eghbalnia HR, Gryk MR, Hoch JC, Maciejewski MW, et al. Merging NMR Data and Computation Facilitates Data-Centered Research. Frontiers in Molecular Biosciences. 2022;8. doi: 10.3389/fmolb.2021.817175 35111815 PMC8802229

[34] Jung Y-S, Hyeon J-S, Hwang G-S. Software-assisted serum metabolite quantification using NMR. Analytica Chimica Acta. 2016;934:194–202. doi: 10.1016/j.aca.2016.04.054 27506360

[35] Mckay RT. How the 1D-NOESY suppresses solvent signal in metabonomics NMR spectroscopy: An examination of the pulse sequence components and evolution. Concepts in Magnetic Resonance Part A. 2011;38A(5):197–220. doi: 10.1002/cmr.a.20223

[36] Huang Y, Yang C, Xu X-f, Xu W, Liu S-w. Structural and functional properties of SARS-CoV-2 spike protein: potential antivirus drug development for COVID-19. Acta Pharmacologica Sinica. 2020;41(9):1141–9. doi: 10.1038/s41401-020-0485-4 32747721 PMC7396720

[37] Haque SKM, Ashwaq O, Sarief A, Azad John Mohamed AK. A comprehensive review about SARS-CoV-2. Future Virology. 2020;15(9):625–48. doi: 10.2217/fvl-2020-0124 33224265 PMC7664148

[38] Shang J, Wan Y, Luo C, Ye G, Geng Q, Auerbach A, et al. Cell entry mechanisms of SARS-CoV-2. Proceedings of the National Academy of Sciences. 2020;117(21):11727–34. doi: 10.1073/pnas.2003138117 32376634 PMC7260975

[39] Grove J, Marsh M. The cell biology of receptor-mediated virus entry. J Cell Biol. 2011;195(7):1071–82. Epub 2011/11/28. doi: 10.1083/jcb.201108131 . 22123832 PMC3246895

